# The Treatment of Cancer of the Cervix Uteri

**Published:** 1914-05-09

**Authors:** 


### THE TREATMENT OF CANCER OF THE CERVIX UTERI.

### Wertheim's Operation, the Bumm and Werder Methods.

Cancer of the cervix of the uterus is a fearfully common disease, and one over which modern medical skill has by no means yet achieved the victory. It is true that the hope of cure to-day for any given patient who suffers from this form of malignant disease is greater—much greater—than it has ever been before; but it is also true that the percentage of cures is still not very large. One cause of the improvement in the statistics in late years is the distinctly earlier date at which the diagnosis is nowadays made on the average. Women do not so frequently put off seeking advice when the initial symptoms of this terrible malady are displayed; and practitioners do not so frequently fail to diagnose the disease when it is submitted to them in its early stages. At the same time there is ample room for further improvement in respect of both these elements of immediate recognition, so indispensable for successful treatment.

So far surgery holds the field as the main hope of cure for such cases of cancer of the cervix as are diagnosed before dissemination has taken place, and before lateral spread has involved structures which are irremovable. Not many years ago surgeons were content to amputate the diseased cervix, clear of the visible growth, through the vagina. The very poor end-results of this operation induced them to adopt the more extensive and radical procedure of vaginal hysterectomy. Again the results were less favourable than had been hoped, and abdominal hysterectomy came into favour. Still the proportion of operable cases was painfully small, and the percentages of cures in those cases was also small.

Then came Wertheim's bold and far-reaching operation, which not only made it possible to submit to operation a much greater proportion of patients than before, but also afforded a much larger chance of complete permanent success. For some years now Wertheim's operation has held the field, and its results far surpass anything that was possible before its introduction. Nevertheless, they are far from ideal. The operation has a very high mortality, even in the hands of the most skilled surgeons: parenthetically it is no operation for any but those who are expert and experienced surgeons. Sir J. Bland Sutton, who collected statistics for 1910 from all the London hospitals where this operation was done, found the operative mortality to be 16 per cent. It is, probably, slightly lower now; but it is doubtful whether, at the best, it is less than 10 per cent.

The greater part of this mortality is due to sepsis; and the sepsis is due to the fact that during the operation a large cavity—the vagina—swarming with septic organisms from the ulcerating surface of the cancer, is placed in immediate communication with extensively dissected pelvic tissues, wherein are numerous cut vessels occluded by ligatures. Septic thrombosis, embolism, peritonitis, and surgical shock from the severity of the operation, are the main causes of this mortality. Since the general recognition of the influence of sepsis on patients after Wertheim's operation, many surgeons have devoted themselves to its elimination. Preliminary disinfection of the vagina is one obvious precaution. Some rely on swabbing out that passage with antiseptics just before the vagina is cut—in the middle of the operation, that is to say. Some curette the growth and swab both it and the vagina with iodine just before they commence the abdominal section. Some cauterise the cervix thoroughly, either as the first stage of the operation or a few days beforehand. Some cauterise the cut edges of the vagina, through the abdominal wound, directly the uterus has been removed.

In all these ways surgeons are striving to reduce the main defect of Wertheim's operation—its high primary mortality. Exactly what percentage of cures may be expected from the operation depends partly on the system of selection of cases. Some authors speak of 50 per cent. of permanent cures, a figure which is undoubtedly far too high. By operating upon very early cases alone, a 25 per cent. rate is perhaps not impossible; but we doubt whether any surgeon who gives his patients the benefit of the doubt as to operability gets a better percentage of eventual cure than 15; and many get nowhere near that figure.

This is, of course, not a state of things with which surgery can be content. Whether it represents the limits of benefit from Wertheim's operation is difficult to say; but it is noticeable that alternative methods are being experimented with. Bumm, it is reported, has given up Wertheim's operation in favour of treatment by diathermy and radium. This famous Continental surgeon's statistics will be watched with great interest, though it is clear that conclusive results cannot be reported for some time to come, on account of the need for tracing the after-history of patients for five years at least. Suffice it that the other leading gynæcological surgeons in Europe have not yet seen fit to follow his example.

Another surgeon, Werder, uses the cautery altogether for removing the growth. He curettes the cervix to begin with, and then cauterises it with the galvano-cautery. He then seizes the cervix with a tenaculum, pulls it down, and sears through the vagina clear of the growth with the iron at a dull-red heat. Care is taken in front not to burn through into the bladder; but behind he opens right through into Douglas's pouch. The vagina is then packed full of gauze. The abdomen is next opened in the usual way, and the peritoneum reflected from the uterus anteriorly, as in Wertheim's operation. Then the cautery is once more resorted to; the infundibulo-pelvic ligaments are burnt through, and then the broad ligaments and parametrium. The uterine arteries are sealed as a rule; but if they bleed they are ligatured. Thus the uterus and the growth are literally burnt out, and it is claimed that accidents to ureters and bladder are no more frequent than in Wertheim's operation. The glands are not removed in this operation of Werder's, which is much less severe than Wertheim's, and has a much lower mortality (5 per cent.). The percentage of cures in cases of five years' standing is stated to be 18. It remains to be seen what the verdict of gynæcologists is upon these novel methods. At least there is a spirit of enterprise prevailing, which is the surest guarantee of continued progress. Possibly the next great advance will be brought about by radium therapy; though hitherto, in spite of a few sensational successes, the results of this form of treatment have been conflicting, and, on the whole, disappointing.

